# Tumor Necrosis Factor-α (TNFα) Stimulate Triple-Negative Breast Cancer Stem Cells to Promote Intratumoral Invasion and Neovasculogenesis in the Liver of a Xenograft Model

**DOI:** 10.3390/biology11101481

**Published:** 2022-10-09

**Authors:** Harini Narasimhan, Francesca Ferraro, Andreas Bleilevens, Ralf Weiskirchen, Elmar Stickeler, Jochen Maurer

**Affiliations:** 1 Department of Obstetrics and Gynecology, University Hospital Aachen, D-52074 Aachen, Germany; 2 Institute of Molecular Pathobiochemistry, Experimental Gene Therapy and Clinical Chemistry (IFMPEGKC) RWTH University Hospital Aachen, D-52074 Aachen, Germany; 3 Center for Integrated Oncology (CIO), Aachen, Bonn, Cologne, Düsseldorf (ABCD), Venusberg-Campus 1, 53127 Bonn, Germany

**Keywords:** triple-negative breast cancer, cancer stem cells, TNFα, EMT, seed-and-soil theory

## Abstract

**Simple Summary:**

In this study, we investigated the effect of TNFα on primary triple-negative breast cancer (TNBC) stem cells to understand the role of TNFα in vascular attraction in the tumor-microenvironment and pre-metastatic niche formation in the liver.

**Abstract:**

TNBC represents the most aggressive breast cancer subtype. Although cancer stem cells (CSCs) are a minor fraction of all cancer cells, they are highly cancerous when compared to their non-stem counterparts, playing a major role in tumor recurrence and metastasis. Angiogenic stimuli and the tumor environment response are vital factors in cancer metastasis. However, the causes and effects of tumor angiogenesis are still poorly understood. In this study, we demonstrate TNFα effects on primary triple-negative breast cancer stem cells (BCSCs). TNFα stimulation increased the mesenchymality of BCSCs in an intermediate epithelial-to-mesenchymal transition (EMT) state, enhanced proliferation, self-renewal, and invasive capacity. TNFα-treatment elicited BCSC signaling on endothelial networks in vitro and increased the network forming capacity of the endothelial cells. Our findings further demonstrate that TNFα stimulation in BCSCs has the ability to instigate distinct cellular communication within the tumor microenvironment, inducing intra-tumoral stromal invasion. Further, TNFα-treatment in BCSCs induced a pre-metastatic niche through breast-liver organ crosstalk by inducing vascular cell adhesion molecule-1 (VCAM-1) enriched neovasculogenesis in the liver of tumor-bearing mice. Overall, TNFα is an important angiogenic target to be considered in breast cancer progression to attenuate any angiogenic response in the tumor environment that could lead to secondary organ metastasis.

## 1. Introduction

Despite recent advances in therapeutic development, breast cancer (BC) is still the leading cause of cancer-related mortality among women, worldwide [1]. In particular, TNBC, lacking estrogen receptors (ER), progesterone receptors, and HER2 receptors, is considered to be the most aggressive of the molecular BC subtypes [2]. Accumulating evidence attributes this aggressiveness of TNBC to the presence of CSCs, which are supposed to play a vital role in therapy resistance, metastasis, and tumor recurrence [3,4,5]. This is attributed to their ability to re-generate the stem cell phenotype (self-renewal) [6,7,8]. Additionally, the presence of CSCs contributes to TNBC aggressiveness by initiating the EMT state of the tumor cell [5,9].

Chronic inflammation in BC plays a role in initiation, development, and survival outcomes [10,11]. TNFα is considered to be one of the most important inflammatory cytokines among the numerous cytokines secreted in the tumor milieu [12,13]. It has multiple biological functions and is, therefore, considered to be a pleotropic cytokine [14]. In addition, this cytokine is produced by diverse cell types, mediating cellular communication and infiltration within the tumor microenvironment [11]. Over the last decade, TNFα has been found to be involved in tumor cell proliferation, aggressiveness, EMT, tumor recurrence, and metastasis [15].

The mechanisms triggering angiogenic and metastatic cascades in the tumor microenvironment are not completely understood. The alteration of adhesive properties of tumor cells mediated by changes in the expression of cell adhesion molecules is one of the most prominent features of the metastatic cascade [16]. TNFα has a pivotal role in the activation of VCAM-1 (CD106), a glycoprotein that is predominantly expressed by endothelial cells [17]. VCAM-1 on endothelial cells has the capacity to capture tumor cells, hinting at a mechanism for metastatic spread [18,19]. Increasing evidence suggests that VCAM-1 expression in BC is associated with lung, bone, and brain metastasis by promoting angiogenesis and BC survival [20,21].

BC is a diversified disease consisting of various subtypes, with each having distinct characteristics, survival rates, and therapies. TNFα is a pleotropic cytokine with reportedly contradictive effects in tumorigenesis which are highly concentration-dependent [22]. Sequencing, serum-profiling and immunohistochemistry studies showed high expression of TNFα leading to metastasis in TNBC patients [23,24]. In addition, animal studies contributing to tumor-promoting roles have been observed in TNBC [25]. Moreover, TNFα gene knockdown is associated with inhibition of cell proliferation and apoptosis in TNBC [26].

On account of their high significance to tumor progression in TNBC, here, we investigated the effect of TNFα on primary epithelial BCSCs from TNBC to determine if this stimulation could have any impact on the tumor microenvironment and increase the proangiogenic phase by inducing a pre-metastatic niche in a secondary organ, in our case, the liver.

## 2. Materials and Methods

### 2.1. Cell Line

Cell lines BCSC1 and BCSC2 were isolated and established as mentioned in our previous study [5,27,28]. The cell lines BCSC1 and BCSC2 were isolated from TNBC individuals who had received chemotherapy. Primary BCSC lines were isolated as mentioned in previous studies by mechanical dissociation and enzymatic digestion in 5 mL of DPBS (Gibco, 70011044, Grand Island, NY, USA) supplemented with 6 units of DNAse I (Machery-Nagel GmbH & Co. KG, 740963, Düren, Germany) and 1 mg of liberase (Roche GmbH, 05466202001, Mannheim, Germany) for 1 h at 37 °C [5,27,28]. The digestion medium was diluted with 10 mL of DPBS and filtered through a cell strainer (40 μm, Falcon, Corning, 352340, Durham, NC, USA). The cell pellet was washed with mammary epithelial basal medium MEBM (Lonza, CC-3151, Basel, Switzerland), after centrifugation at 200 ×g for 5 min. Red blood cells were removed using 2 mL of ACK Lysis-buffer (Gibco, A1049201, Grand Island, NY, USA). The suspension was filled with 6 mL of MEBM and centrifuged at 200 ×g for 5 min. The pellet was resuspended in 1 mL of MEBM and filtered through a 40 μm strainer. Following centrifugation at 200 ×g for 5 min, the remaining cell pellet was suspended in MSC medium. Next, 2 × 10^4^ cells in 200 μL of a 1:1 mixture of mammary stem cell (MSC) medium and Matrigel (ice cold, Corning, 354230, Bedford, MA, USA) were plated per well in a 24-well low attachment plate (Corning, 3473, Kennebunk, ME, USA). After solidification of the Matrigel at 37 °C for 30 min, 500 μL of MSC medium was added to each well. The cells were cultured at 37 °C under low oxygen conditions (3% O2, 5% CO2, 92% N2). Three-dimensional cells, stably proliferating cells were cultured and expanded in 2D. All primary BCSC lines were isolated in 2014 and authenticated by the high-throughput multiplex human cell authentication test (MCA) developed at the German Cancer Research Center (DKFZ) in 2016.

### 2.2. Cell Culture and Medium

BCSC1 and BCSC2 were cultured in MSC medium prepared and cultured as established in our previous study [5,27,28]. The MSC medium consists of (MEBM) (Lonza, CC-3151), supplemented with 1× B27 (Gibco, 17504-001, Grand Island, NY, USA), 1× amphotericin B (Gibco, 15290-026, Grand Island, NY, USA), 1× penicillin–streptomycin (Gibco, 15140-122, Grand Island, NY, USA), epidermal growth factor (20 ng/mL, PeproTech, AF-100-15, ) Rocky Hill, NJ, USA, heparin (4 μg/mL, Sigma-Aldrich, H3149, Taufkirchen, Germany), fibroblast growth factor (20 ng/mL, PeproTech, AF 100-18B, Cranbury, NJ, USA), gentamicin (35 μg/mL, Gibco, 15750-045, Paisley, UK), and rho kinase inhibitor (500 nmol/L, Calbiochem Sigma-Aldrich, 555552, Darmstadt, Germany).

### 2.3. Cell Proliferation Assay

For cell proliferation assay, 96 well plates (Falcon, Corning, 353072, Durham, NC, USA) were used. The well plate was precoated with 2% of Matrigel in a volume of 50 µL of MSC medium. Then, 0.3 × 10^4^ BCSC1 and BCSC2 RFP tagged cells were seeded in 100 µL after the solidification of Matrigel. After 24 h, the target group of cells received 150 µL of fresh MSC medium with 100 ng/mL TNFα (Peprotech, 300-01A, Cranbury, NJ, USA) daily for 5 days. The proliferation was observed in the IncuCyte^®^ S3 Live-Cell Analysis System (Sartorius, Ann Arbor, MI, USA). The dosage and duration of the TNFα stimulation on BCSCs was concluded from a pilot study (data not shown).

### 2.4. 3D Sphere Forming Assay

BCSCs were treated with or without TNFα for 10 days. Then, 0.5 × 10^3^ BCSC1 or BCSC2 cells were seeded in 50% Matrigel in a 1:1 mixture volume of Matrigel and MSC medium in a 96-well ultralow attachment plates (Corning, CLS7007-25EA, Durham, NC, USA). The total number of spheres was counted on day 8. The spheres were imaged using Invitrogen™ EVOS™ FL Auto Imaging System (ThermoFisher, Bothell, WA, USA).

### 2.5. Reverse Transcription and Quantitative PCR

mRNA was isolated from untreated and 10 days TNFα-treated BCSCs using RNeasy Mini Kit (Qiagen, 1038703) initially. Reverse transcription was performed as described in our previous study [5]. The qPCR was performed using the Universal Probe Library (UPL Roche, 04683633001, Mannheim, Germany) system by Roche LightCycler^®^480 to analyze the relative gene expression for BCSC1. iQ™ SYBR^®^ Green Supermix (Bio-Rad Laboratories, 1708880, Hercules, CA, USA) was used to analyze the relative gene expression for BCSC2 due to the discontinuation of the UPL probes by Roche. For both the methods, the normalization was performed using Actin Beta (ACTB). The primers along with the sequences are listed in the table (Table S1). For VCAM-1 gene expression, PrimePCR™ SYBR^®^ Green Assay VCAM-1 (Bio-Rad Laboratories, qHsaCID0016779, Hercules, CA, USA) was used for both the cell lines. For NF-κB and MAPK signaling pathway, PrimePCR™ SYBR^®^ Green Assay Nfkb1 (Bio-Rad Laboratories, qMmuCED0047222, Hercules, CA, USA) and PrimePCR™ SYBR^®^ Green Assay MAPK8 (Bio-Rad Laboratories, qMmuCED0045823, Hercules, CA, USA) was used, respectively.

### 2.6. Protein Isolation

The cells were cultured and treated with TNFα for 10 days as mentioned above. The isolation of the protein was performed according to our previous study [5]. The concentration of the protein was determined according to the instructions provided by the DC™ Protein Assay Kit II (Bio-Rad Laboratories, 5000112, Hercules, CA, USA).

### 2.7. Western Blot

Mini-PROTEAN^®^ TGX™ Precast Protein Gels (Bio-Rad Laboratories, 456-9036, Hercules, CA, USA) was loaded with 25 µg of protein lysate consisting of Laemmili buffer (Bio-Rad Laboratories 161-0747, Hercules, CA, USA)and 2-Mercaptoethanol (Sigma Aldrich, M6250, Taufkirchen, Germany). Precision Plus Protein Dual Color Standards (Bio-Rad Laboratories, 161-0374, Hercules, CA, USA) was used as the standard ladder for reference. The blotting was performed at 300 W, 0.5 A for 1 h using Bio-Rad wet blotting system. After gel electrophoresis, the blot was transferred to PVDF membrane using Trans-Blot Turbo Transfer System from Bio-Rad. The membrane was blocked using 5% BSA for 1 h. The listed antibodies (Table S2) were diluted with blocking solution and incubated at 4 °C overnight. The membranes were washed with TBST the following day. Following washing, the membranes were incubated with the secondary antibody diluted in 5% BSA for 1 h at room temperature. The membranes were washed and detected using the Miracle Star ™ ECL Reagent (iNtRON Biotechnology, 16028, Gyeonggi-do, Korea). Fusion SL (Vilber Lourmat GmbH, Eberhardzell, Germany) was used for detecting the chemiluminescent signal.

### 2.8. Cell Invasion Assay

CytoSelect™ 24-Well Cell Invasion Assay, Basement Membrane (Cell Biolabs, CBA-110, San Diego, CA, USA) consisting of 8-μm pores inserts with basement membrane was used. The inserts were rehydrated with serum free MSC medium. Then, 1.8 × 10^5^ cells were resuspended in MSC medium and seeded into each insert which was placed on a well consisting of 500 µL of MSC medium with 10% FBS for 48 h. The well plate was incubated at 3% O_2_, 5% CO_2_, 92% N_2_ atmosphere. The invasive cells were imaged and quantified with Echo Rebel (Discover Echo, San Diego, CA, USA).

### 2.9. Conditioned Medium

To prepare the conditioned medium, the cells were cultured and treated with TNFα as mentioned previously. The control cells received medium change twice in 5 days. On day 11, the medium from both the groups was centrifuged distinctly at 200 × g for 3 min. The supernatants were aliquoted and used for further experiments.

### 2.10. Tube Formation Assay

For tube formation assay, 50 µL of Matrigel (Corning, 354230, Bedford, MA, USA) was precoated on 96 well plates (Falcon, Corning, 353072, Durham, NC, USA). Next, 2 × 10^4^ HUVECs mixed with 100 µL of CM was seeded in each well. For positive control, fresh MSC medium was used. To ensure the effect of network formation was solely not dependent on TNFα, fresh MSC medium with 100 ng/mL TNFα was used as a control. For the negative control, depleted medium was used. The tube formation assay was monitored in the IncuCyte^®^ S3 Live--Cell Analysis System (Sartorius, Ann Arbor, MI, USA).

### 2.11. Orthotopic Breast Cancer Xenografts

The orthotopic transplantation was performed as described in our previous study [5,28]. BCSCs tagged with Luciferase either untreated or TNFα-treated for 10 days was used for transplantation. NOD/SCID females (4–5 weeks old) were anesthetized using an isoflurane inhalator. A small sagittal incision (no longer than 1.0 cm) on the shaved and sterilized abdomen allowed access to the mammary gland No. 4 on both sides. Indicated numbers of BCSCs were mixed with 1*10^6^ irradiated fibroblasts (newborn human foreskin fibroblasts (NuFF), p11, GlobalStem, Rockville, MD, USA) and suspended in a 1:1 mixture of Matrigel (Corning, 354230, Durham, NC, USA) and MSC medium in a total volume of 40 µL per gland. The mixture was injected into the mammary fat pad of the No. 4 gland on both sides of the animal. Each transplant was localized distal to the lymph node in the gland. Surgical incisions were sealed by suturing with a 5/0 thread (Ethicon, Z995, New Brunswick, NJ, USA). Animals were monitored twice weekly for weight and tumor growth, which was determined by caliper measurement. Tumor volumes were calculated using the following formula: 4/3 x π x r3.

### 2.12. Immunohistochemistry

The IHC analysis was performed as per our previous study [5,28]. Xenograft tumor tissue specimens were fixed in 10% formalin and embedded in paraffin. Two-μm-thick paraffin-embedded tissue sections were mounted onto glass slides. All slides were stored for two days at 58 °C in a drying chamber, subsequently deparaffinized using xylene and hydrated with ethanol. The tumor tissues were stained with the primary antibodies listed (Table S3).

### 2.13. Elastica Van Gieson (EVG) Staining

To determine the presence of stromal proteins, EVG staining was performed using Elastic Stain Kit (Abcam, ab150667, Cambridge, UK) according to the manufacturer’s protocol. The images were captured using Echo Rebel (Discover Echo, San Diego, CA, USA). The quantification was performed using ImageJ software.

### 2.14. Statistical Analysis

Results are expressed as mean ± SEM. Two group data were compared using two-tailed, unpaired Student’s *t*-test for all assays. Statistical significance for growth curve was determined using 2-way ANOVA with Sidak’s multiple comparison test. Tube formation assay was analyzed using 1-way ANOVA using Sidak’s multiple comparison test. *p*-values of <0.05 were considered significant.

## 3. Results

### 3.1. TNFα Induces EMT, Such as Phenotype with Increasing Self-Renewal Capacity in TNBCSCs

As described in our previous study, we established four BCSC lines (*1–4*), which were isolated from four individual breast cancer patients belonging to the TNBC subtype [28]. The tumorigenicity of derived cells was examined using limiting dilution transplantation experiments. These cell lines recapitulate the phenotype of the original patient’s tumor when transplanted into an immunocompromised mouse, phenocopying the original patient’s tumor cytoarchitecture and have been characterized as BCSCs [28].

**Figure 1:**
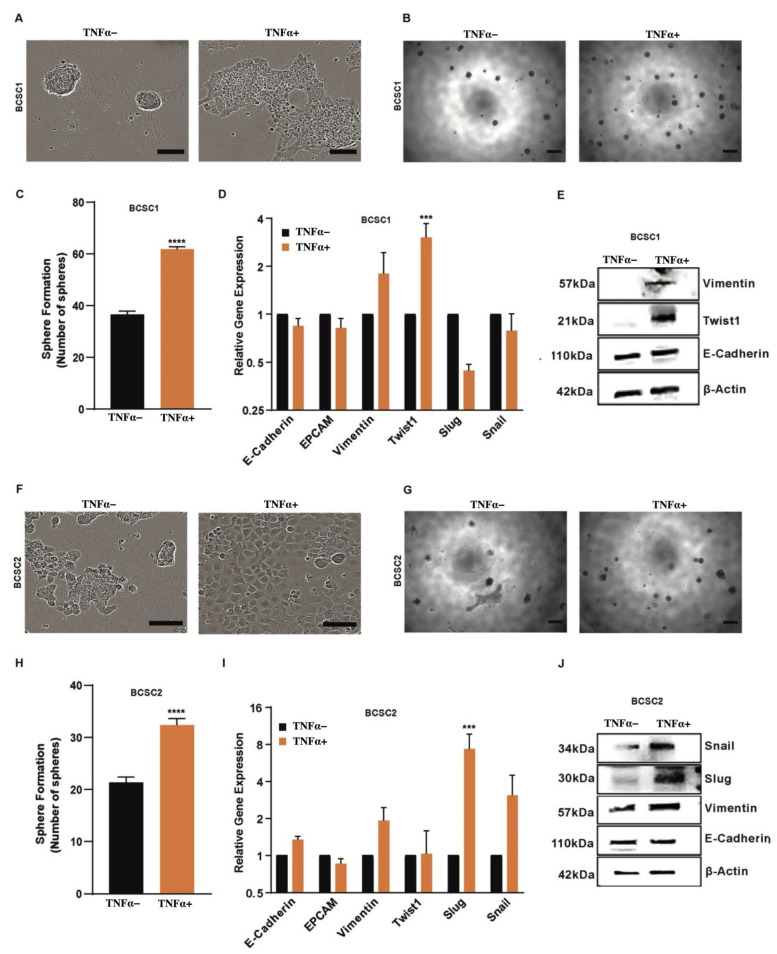
Effect of TNFα stimulation in BCSCs. (**A**) Morphology of untreated and TNFα-treated BCSC1. Scale bars, 20 µm. (**B**) Sphere forming assay of untreated and TNFα pretreated BCSC1 (*n* = 3). Scale bars, 37.5 µm. (**C**) Graphical representation of data showing the count of spheres. Data represents mean ± SEM; **** *p* < 0.0001 by two-tailed, unpaired Student’s *t*-test. (**D**) Relative gene analysis of indicated epithelial-to-mesenchymal transition (EMT) markers in untreated and TNFα-treated BCSC1 (*n =* 3). Data represents mean ± SEM; *** *p* < 0.001, by 2-way ANOVA. For the untreated group, data represents no error bar as data was normalized to 1 to denote the fold change. (**E**) Western blot analysis of EMT markers in BCSC1. (**F**) Morphology of untreated and TNFα-treated BCSC-2. Scale bars, 20 µm. (**G**) Sphere forming assay of untreated and TNFα-treated BCSC2 (*n =* 3). Scale bars, 37.5 µm. (**H**) Graphical representation of data showing the count of spheres. Data represents mean ±S EM; **** *p* < 0.0001 by two-tailed, unpaired Student’s *t*-test. (**I**) Relative gene analysis of indicated EMT markers in untreated and TNFα-treated BCSC2 (*n =* 3). Data represents mean ± SEM; *** *p* < 0.001, by 2-way ANOVA. For the untreated group, data represents no error bar as data was normalized to 1 to denote the fold change. (J) Western blot analysis of EMT markers in BCSC2.

It has been reported that TNFα can induce EMT in breast cancer cells [29]. We showed, in our previous study, that BCSCs have an intermediate EMT phenotype [5]. Hence, we supposed TNFα stimulation of BCSCs can induce a more mesenchymal phenotype. In order to observe morphological changes, BCSCs were cultured with TNFα in the medium for 10 days. Indeed, treatment with TNFα altered the morphology of BCSCs significantly after three days. TNFα-treated BCSC1 (Figure 1A, right panel) and BCSC2 (Figure 1F, right panel) showed loose epithelial clusters while the untreated BCSC1 (Figure 1A, left panel) and BCSC2 (Figure 1F, left panel) cells maintained a cobblestone-like epithelial structure with tight cell-to-cell contacts.

RT-qPCR analysis showed a significant increase in mesenchymal marker TWIST1 and a moderate upregulation of Vimentin in BCSC1 (Figure 1D), while in BCSC2, significant expression of SLUG along with minor expression of Vimentin and SNAIL was observed (Figure 1I). The switch to a more mesenchymal phenotype was also reflected at the protein level. The mesenchymal markers that showed marginal or significant upregulation in RT-qPCR were analyzed for protein expression along with the epithelial marker E-Cadherin. Western blot analysis of BSCS1 (Figure 1E) and BCSC2 (Figure 1J) confirmed the increase in mesenchymal markers, while E-Cadherin expression remained constant, confirming an intermediate EMT phenotype. The densitometric analysis of the blot was performed and a subsequent graph depicting the relative fold change of the protein was measured (Figure S1A–F).

BCSC1 and BCSC2 showed both luminal and myoepithelial keratin expression, thereby characterizing the bi-potential stemness of these cells [5]. To understand if TNFα influences this stemness, we performed immunofluorescence staining of the cells with keratin markers K5/K8 (Table S4). We detected an increase in double positive K5/K8 expression in BCSCs with TNFα treatment, hinting at an increased stemness of TNFα-treated BCSCs (Figure S2A–D). Additionally, functional mamosphere assays showed a significant increase in the sphere formation of BCSC1 (Figure 1B,C) and BCSC2 (Figure 1G,H) treated with TNFα.

### 3.2. TNFα Enhances Proliferation and Invasion in BCSCs

**Figure 2:**
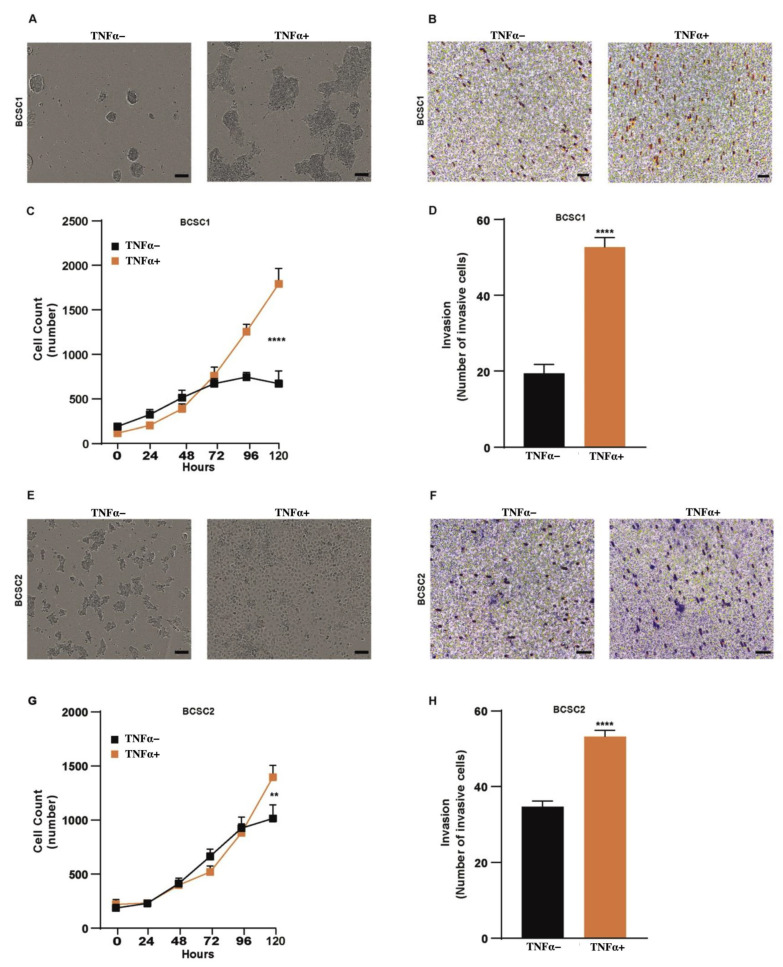
Proliferation and invasion abilities of BCSCs upon TNFα stimulation. (**A**) Cell proliferation assay: BCSC1 were either untreated or treated with 100 ng/mL of TNFα to measure the confluence of the cells for 5 days (*n =* 3). Scale bars, 15 µm. (**B**) Cell invasion assay: BCSC1 untreated or treated with TNFα for 10 days was examined for invasive capacity. Scale bars, 15 µm. (**C**) Graphical representation of BCSC1 cell proliferation. Data represents mean ± SEM; **** *p* < 0.0001 by 2-way ANOVA. (**D**) Data in graph represents number of invasive cells and between the two experimental groups (*n =* 3). Data represents mean ± SEM; **** *p* < 0.0001 by two-tailed, unpaired Student’s *t*-test. Both BCSC1 and BCSC2 were analyzed under the same experimental setup. (**E**) Cell proliferation assay of BCSC2. Scale bars, 15 µm. (**F**) Invasion assay of BCSC2. Scale bars, 15 µm. (**G**) Graphical representation of BCSC2 cell proliferation. Data represents mean ± SEM; ** *p* < 0.01 by 2-way ANOVA. (**H**) Data in graph represents number of invasive cells and between the two experimental groups (*n =* 2). Data represents mean ± SEM; **** *p* < 0.0001 by two-tailed, unpaired Student’s *t*-test.

There has been evidence that TNFα increases proliferation and invasion of cancer cells, thereby promoting tumor progression and cancer metastasis [14,30]. We examined the proliferation of BCSCs under TNFα treatment using automated microscopy. In the presence of TNFα, there was a steady increase in the expansion of BCSC1 (Figure 2A,C) and BCSC2 (Figure 2E,G) when compared to the untreated cells.

Next, we wanted to investigate the invasive capacity of BCSCs. TNFα-treated BCSC1 (Figure 2B,D) and BCSC2 (Figure 2F,H) showed an increase in matrix invasion. Furthermore, TNFα treatment increased the migratory abilities of BCSCs, which was observed in a scratch wound assay (Figure S3A,D). TNFα-treated BCSC1 and BCSC2 showed increased relative wound density and wound confluence (Figure S3B,C,E,F).

### 3.3. TNFα-Treated BCSCs Secrete Factors to Increase Network Formation of the Endothelial Cells In Vitro

**Figure 3:**
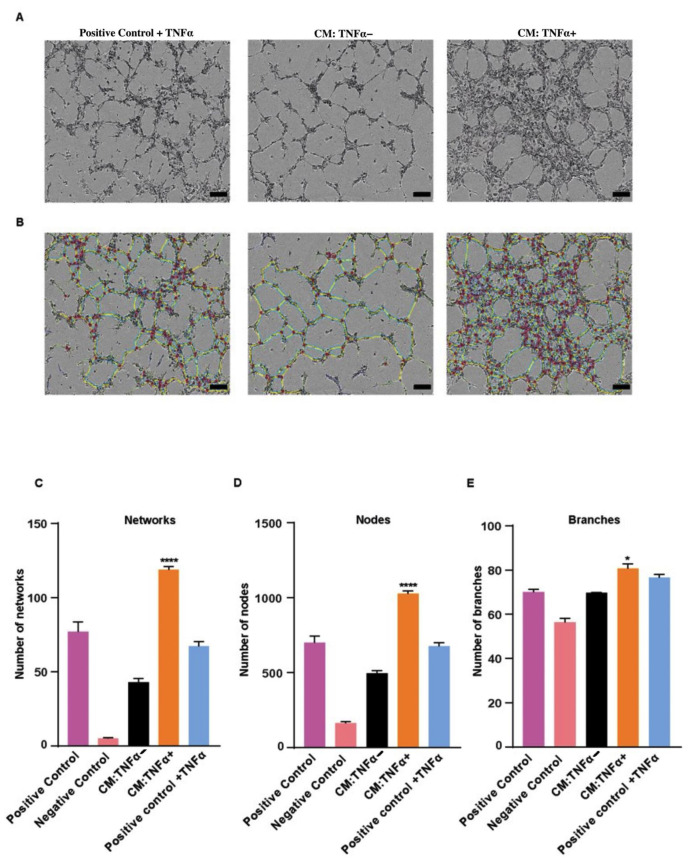
Endothelial cell network forming capacity. To understand the capacity of network formation, fresh MSC medium with TNFα was used as a positive control, depleted medium was used as a negative control and conditioned media (CMs) from untreated and 10 days TNFα-treated BCSC1 were used. (**A**) TNFα-CM from BCSC1 promoted the tube formation of HUVECSs in vitro. Scale bars, 15 µm. (**B**) Representative images depicting the quantitative analysis of the tube formation. Data analysis was performed with Image-J Plugin angiogenesis analyzer. Blue color indicates the networks. Small circles indicate the nodes. Green color indicates the branches. (**C**–**E**) Graphical data demonstrating the number of networks, nodes, and branches (*n =* 3). Data represents mean ± SEM; * *p* < 0.05, **** *p* < 0.0001 by 1-way ANOVA.

To analyze whether TNFα is involved in vascular attraction by BCSCs, we performed tube formation assays with human umbilical vein endothelial cells (HUVECs) in vitro. BCSC1 and BCSC2 cells were treated with or without TNFα for 10 days. Conditioned medium (CM) from these cultures was added to HUVECs to observe the formation of networks. The HUVECs established solid networks 3 h following the addition of CM obtained from BCSC1 cells. An MSC medium with added TNFα was used as a control. The number of nodes, networks, and branches generated by the HUVECs was increased with CM obtained from TNFα-treated BCSC1 when compared to other controls (Figure 3A,B).

A mean of 118 networks and 1048 nodes were quantified with TNFα-treated CM, while a mean of 42 networks and 495 nodes were quantified with untreated CM (Figure 3C,D). The aggregate number of branches showed a moderate increase in the TNFα-treated group (Figure 3E). The HUVECs showed no significant difference following the addition of the CM obtained from BCSC2 cells (Figure S4A–E).

### 3.4. TNFα Increases Tumor Growth of BCSCs

**Figure 4:**
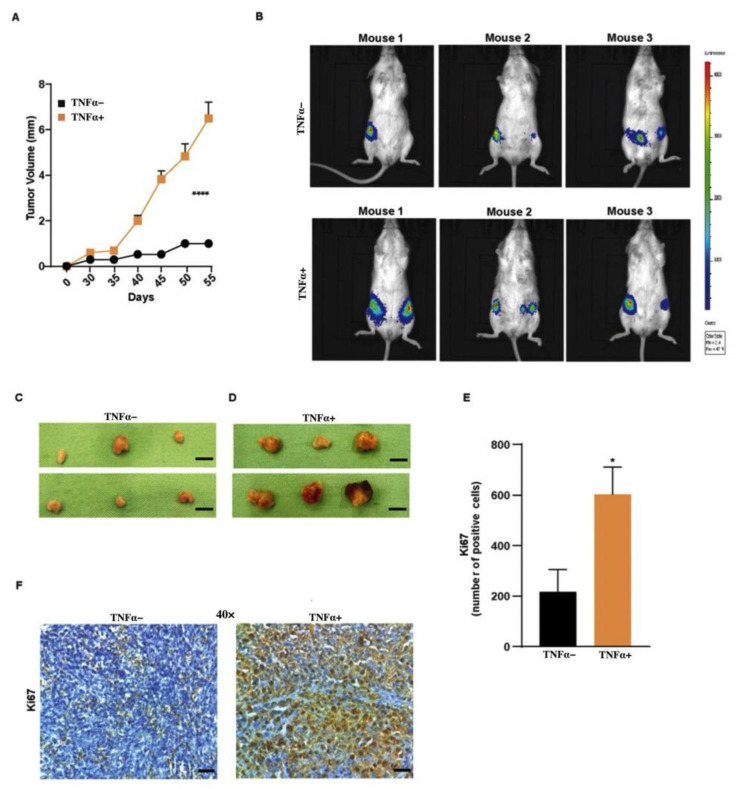
Effects of TNFα in vivo. Untreated or TNFα-treated luciferase-tagged BCSC2 were injected orthotopically in both mammary pads of NOD-SCID mice. (**A**) Tumor growth curve over time (*n* = 6/group). Data represents mean ± SEM; **** *p* < 0.0001 by 2-way ANOVA. (**B**) Representative bioluminescent images of the tumor-bearing mice 6.5 weeks post transplantation. (**C**,**D**) Excised tumors of the mice from the two experimental group (*n =* 6/group). Scale bars, 5 mm. (**E**) Mice that received TNFα-treated BCSC2 showed increased Ki67-positive cells (*n =* 5/group). (**F**) Ki67 staining of the tumor tissues (*n =* 5/group). Scale bars, 7.5 µm. Data represents mean ± SEM; * *p* < 0.05 by two-tailed, unpaired Student’s *t*-test.

To investigate the effect of TNFα in vivo, we performed the gold standard assay of orthotopic transplantation of BCSCs in NOD-SCID mice. BCSC1 or BCSC2 tagged with luciferase were treated with TNFα for 10 days. Then, 1 × 10^5^ cells were orthotopically injected into both mammary pads of the mice. A bioluminescence signal was observed at 3.5 and 6.5 weeks post-transplantation for mice that received BCSC1 (Figure S5B) and BCSC2 (Figure 4B) cells, respectively. Mice that received TNFα-treated BCSC2 cells showed dramatically accelerated tumor growth (Figure 4A,D) when compared to untreated cells (Figure 4A,C). Using Ki67 immunohistochemistry, we detected a strong increase in the number of proliferating cells in the TNFα-treated BCSC2 tumors (Figure 4E,F). Tumors from BCSC1 cells, on the contrary, grew at a similar speed and remained similar in Ki67 expression compared to their controls (Figure S5A–E).

To address the tumor growth difference between the cell lines, RNA was isolated from the tumors and gene expression analysis was performed to understand the signaling cascade of the two cell lines. Although there was no significance between the untreated and TNFα-treated groups in the signaling pathway, the gene analysis indicated a difference in signaling pathways among the cell lines, with BCSC1 expressing the NF-κB pathway and BCSC2 expressing the MAPK pathway (Figure S6A,B). Therefore, further research is required to acknowledge the significant difference between the downstream signaling cascade and to further characterize the different features and differences of the two BCSCs involved in the represented pathways.

### 3.5. TNFα-Treated BCSC Tumors Develop Fibrotic Septa with Increased Collagen and Elastin Fibers

**Figure 5:**
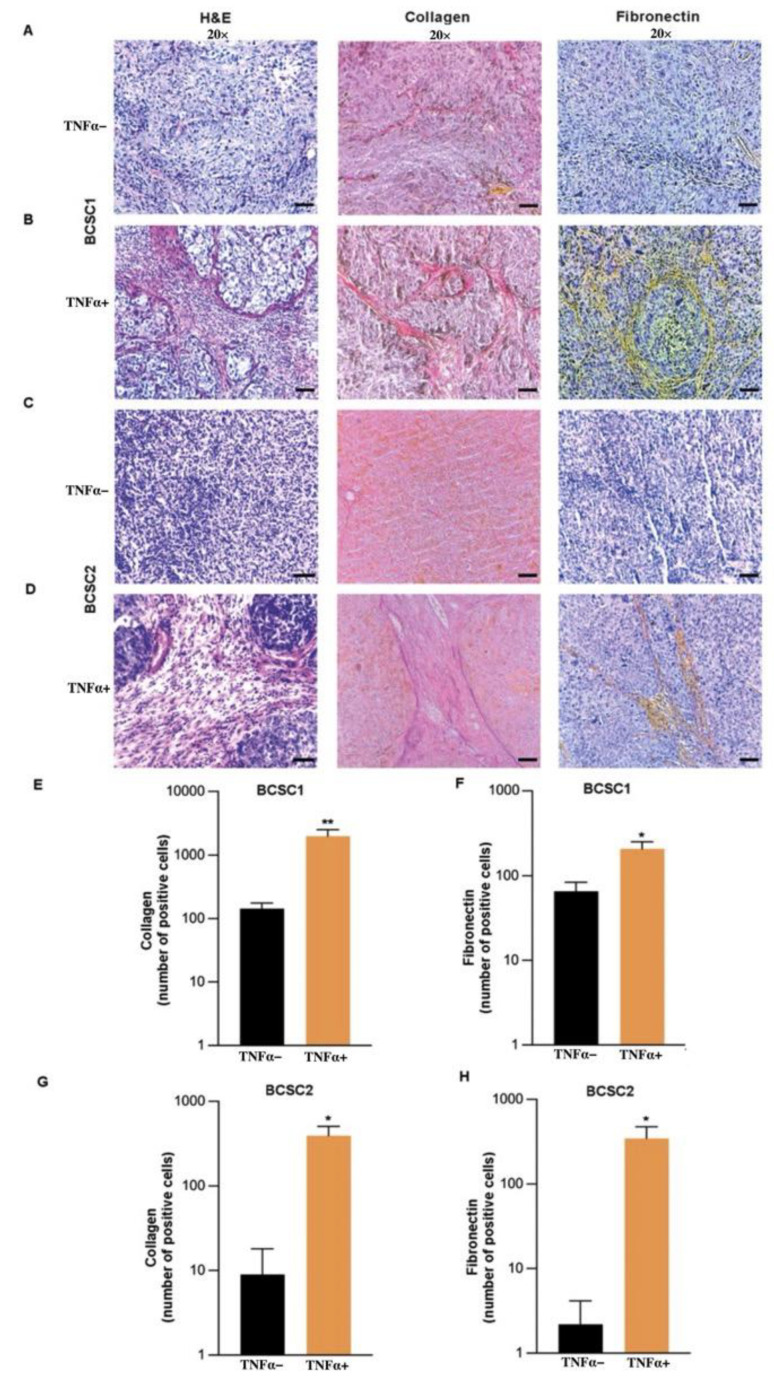
TNFα induces increased fibrotic network formation in vivo. (**A**) H and E staining (*n* = 10/group), EVG staining (*n* = 8/group), and Fibronectin staining (*n* = 5/group) of the tumor tissue from the mice injected with untreated and (**B**) TNFα-treated BCSC1, respectively. Scale bars, 15 µm. (**C**) H and E (*n* = 6/group), EVG (*n* = 4/group) and Fibronectin staining (*n* = 5/group) of the tumor tissue from the mice injected with untreated and (**D**) TNFα-treated BCSC2, respectively. Scale bars, 15 µm. (**E**) Graphical data representing the quantification of collagen fibers and (**F**) fibronectin-positive cells in BCSC1 experimental group and (**G**) collagen fibers. (**H**) Fibronectin-positive cells in BCSC2 experimental group. Data represents mean ± SEM; * *p* < 0.05, ** *p* < 0.01 by two-tailed, unpaired Student’s *t*-test.

H and E staining of tumors from TNFα-treated BCSC1 (Figure 5A,B left panel) or BCSC2 (Figure 5C,D left panel) revealed an increase in fibrotic septa invading the tumors. We speculated that TNFα could be a potential angiogenic stimulus that could promote cell-to-cell crosstalk between the tumor cells and fibroblasts in the tumor microenvironment. As a result of interactions between the cells, the fibroblasts could potentially create an avascular pathway, leading to an intratumoral-stromal invasion. To prove that, elastic Verhoeff–Van Gieson (EVG) staining was conducted to examine collagen involved in the stromal invasion of the primary tumor. The EVG staining revealed an increase in the collagen protein for the TNFα-treated group in both BCSC1 (Figure 5A,B center panel, Figure 5E) and BCSC2 (Figure 5C,D center panel, Figure 5G) cell lines. Furthermore, fibronectin was increased in TNFα-treated group in both BCSC1 (Figure 5A,B right panel, Figure 5F) and BCSC2 cell lines (Figure 5C,D right panel, Figure 5H).

### 3.6. TNFα-Treated BCSCs Leads to Intratumoral Vessel Formation

**Figure 6:**
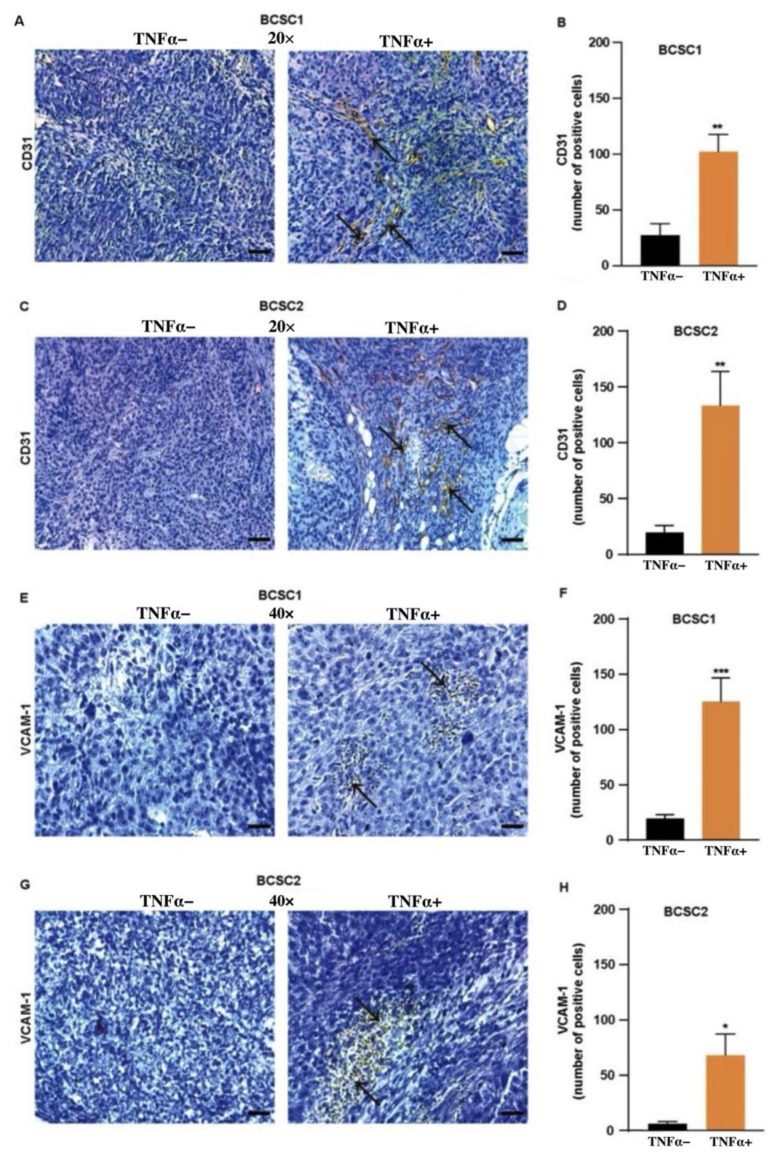
TNFα stimulation increases angiogenic response in vivo. (**A**,**C**) Representative tumor images depicting CD31 staining (brown) in the tumor tissue (blue nuclei) indicating absence or presence of CD31 expressing blood vessels (indicated by arrows) in experimental groups of BCSC1 (*n* = 8/group) and BCSC2 (*n* = 5/group). (**B**,**D**) Quantification showing increase in CD31 positive blood cells in the fibrotic septa in TNFα-treated BCSC1 and BCSC2 group. Scale bars, 15 µm for CD31 and 7.5 µm for VCAM-1. (**E**,**F**) Data showing VCAM-1 (*n* = 6/group)-positive cells (brown/indicated by arrows) between the two treatment groups in BCSC1. (**G**,**H**) Quantification of VCAM-1-positive cells (*n* = 4/group) between the two groups in BCSC2. Data represents mean ± SEM; * *p* < 0.05, ** *p* < 0.01, *** *p* < 0.001 by two-tailed, unpaired Student’s *t*-test.

Studies indicate that intravasation processes occur exclusively within the core of the primary tumor and, therefore, intravasation events are localized to the intratumoral angiogenic vasculature [31]. Other than the intratumoral-stromal invasion, we found that TNFα-treated BCSCs were able to induce intratumoral invasion of the endothelial cells (Figure 6A–D). We hypothesized previously that the cell-to-cell crosstalk between fibroblasts and the tumor cells generates a pathway for intratumoral-stromal invasion. We anticipated that the avascular pathway could attract vasculature in the primary tumor by aiding the endothelial cells towards the primary tumor as an angiogenic response, thus, creating a proangiogenic state within the tumor microenvironment. To verify our hypothesis, tumor tissue samples were stained with the antibody CD31 and quantified. Indeed, the presence of endothelial cells localized in the fibrotic septa was observed in the tumor tissue of the mice that received TNFα-treated BCSC1 (Figure 6A,B) or BCSC2 (Figure 6C,D) cells.

Ectopic expression of VCAM-1 has been observed in breast cancer involving tumor-stromal interactions, angiogenesis, and metastasis [21]. Accordingly, we examined whether TNFα-treated BCSCs would show increased VCAM-1 expression. Interestingly, the expression of VCAM-1 was increased in the TNFα-treated group for both the cell lines (Figure 6E–H). These results clearly indicate that TNFα plays a key role in cell-to-cell interaction between the tumor cells, stromal cells, and endothelial cells, increasing VCAM-1, thereby switching the tumor microenvironment to a more angiogenic state.

### 3.7. TNFα-Treated BCSC Show an Increase in Liver Neovasculogenesis in Mice

**Figure 7:**
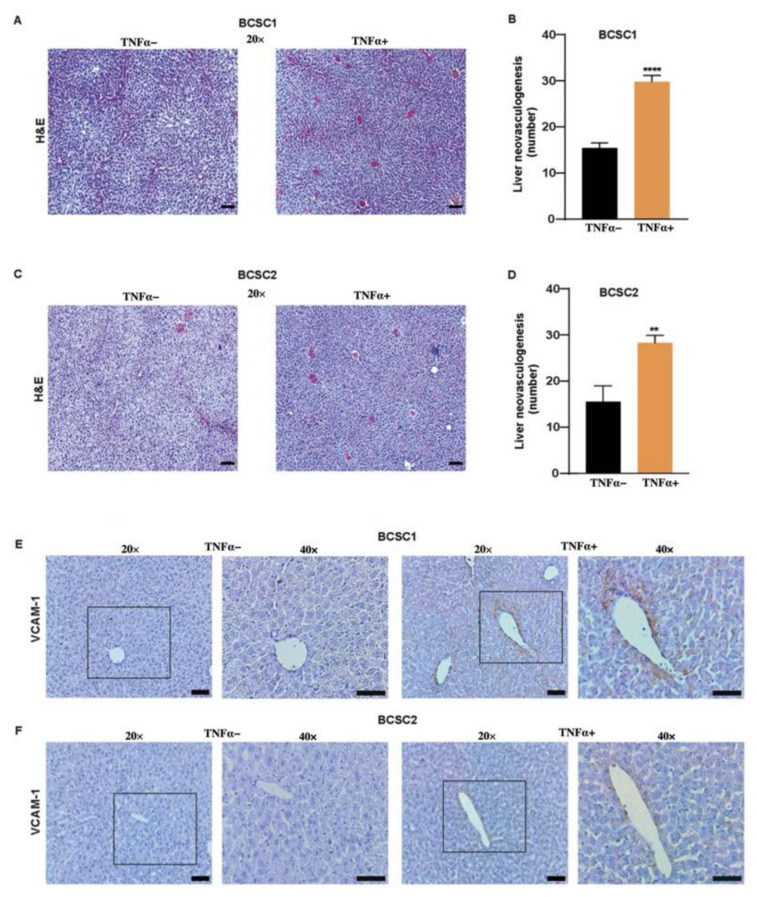
TNFα-treated BCSCs promotes breast-liver organ crosstalk. (**A**) H and E staining of untreated and TNFα-treated BCSC1. Scale bars, 15 µm. (**B**) Quantification of H and E in BCSC1 (*n* = 5/group). (**C**) H and E staining of untreated and TNFα-treated BCSC2. Scale bars, 15 µm. (**D**) Quantification of liver neovasculogenesis in the H and E stains depicted in (**C**) (*n* = 3/group). (**E**) Data depicting the absence of VCAM-1 expression in mice liver tissue in the untreated group (left) (*n* = 5) and VCAM-1 enriched vascularity in TNFα-treated BCSC1 group (right) (*n* = 5). (**F**) VCAM-1 staining in the untreated BCSC2 (left) (*n* = 3) and TNFα-treated BCSC2 (right) (*n* = 3) group. Scale bars, 25 µm and 20 µm. Data represents mean ± SEM; ** *p* < 0.01, **** *p* < 0.0001 by two-tailed, unpaired Student’s *t*-test.

The premetastatic niche is a receptive tissue microenvironment that undergoes several biological changes to organize the metastatic-designated site, priming itself to be the fertile ‘soil’ to receive the ‘seed’ to colonize and enhance the distant organ metastasis [32]. Hence, we wanted to examine if TNFα-treated BCSCs could also have an angiogenic effect on priming the premetastatic niche in the different organs of tumor-bearing mice. Therefore, we analyzed different organs of mice that received untreated or TNFα-treated BCSCs. Apart from the fact that TNFα was involved in cell-to-cell communication in the tumor microenvironment, the inflammatory cytokine had the capacity to induce neovasculogenesis in the liver of NOD-SCID mice. H and E staining and analysis showed increased neovasculogenesis induced in the liver of mice that received TNFα-treated BCSC1 or BCSC2 cells (Figure 7A–D). CD31 staining was performed to confirm the presence of endothelial cells in the liver neovasculogenesis in both cell lines (Figure S7A,B).

We postulate that increased TNFα-treated BCSCs could prime the liver to receive metastatic cells by inducing neovasculogenesis. The presence of VCAM-1 on the endothelial cell is known to promote extravasation and distant organ metastasis [33]. In order to confirm this, mouse liver tissue was subjected to RNA isolation and gene analysis was performed by RT-qPCR. The data showed increased relative gene expression of VCAM-1 in the livers of mice that received TNFα-treated BCSC1 (Figure S7C) or BCSC2 (Figure S7D). Moreover, histological staining showed that neovasculogenesis was endowed with VCAM-1 expression in liver tissue in the TNFα-treated group in comparison to the untreated group in both the cell lines (Figure 7E,F). Although not all animals showed VCAM-1-enriched liver vasculogenesis, an increased number of mice were observed to bear these characteristics (Table S5). These data suggest that TNFα not only has an impact on the cell-to-cell crosstalk, but also has an explicit role in the breast-liver organ crosstalk mediating VCAM-1 expression. This further indicates that TNFα-treated cells prepare a pre-metastatic niche to potentially materialize the ‘seed’ (BCSCs) for preparing the soil (liver) for metastatic cells with VCAM-1 expression.

Taken together, we propose a model where TNBCSCs exposed to the inflammatory cytokine TNFα, can induce EMT and vascular attraction. TNFα can potentially act as an angiogenic stimulus, which leads to distinct cell communication in the tumor microenvironment, inducing VCAM-1 expression. Moreover, this stimulation results in organ crosstalk between the breast and liver, indicating a premetastatic niche in the liver by producing VCAM-1-enriched neovasculogenesis, which could lead to a potential liver metastasis. However, further research is required to understand the deep underlying mechanisms for achieving a successful colonization in the liver.

## 4. Discussion

Other than being one of the major proinflammatory cytokines, TNFα acts as a mediator of cancer-related inflammation in the tumor microenvironment [34,35,36]. The connection between TNFα and BC has been investigated for the past two decades. Although many studies have shown the multifarious effects of TNFα in tumor progression in various cancers [37,38], several questions remain unaddressed with regard to the complex biological signaling of this pleotropic cytokine in the tumor microenvironment.

In the present study, we stimulated the well-established BCSC1 and BCSC2 with TNFα. These cells, being already in a state of intermediate EMT [5], were shifted to a more mesenchymal phenotype showing on the one hand the drive with TNFα to a more mesenchymal phenotype, on the other hand depicting the flexibility of the intermediate EMT state BCSCs reside in. Treatment with TNFα for three days was sufficient to induce morphological differences. Simultaneously, we found upregulation of known mesenchymal markers, Twist1, vimentin and slug, and snail. Twist1 is a regulator of TNFα-induced EMT in MCF-10A and HBL-100 cells [38]. However, in contrast to MCF10A cells, the intermediate character of EMT in BCSC1 and BCSC2 remained, as indicated by the presence of E-Cadherin in both cell lines. The nature of intermediate EMT in BCSCs seems to be rather flexible than absolute. In our previous study, intermediate EMT plasticity of BCSCs was also observed with TGFβ stimulation, which showed a different molecular mechanism in comparison to TNFα stimulation [5].

The ability of self-renewal and multilineage differentiation are the typical characteristics of stem cells and also of BCSCs [5,27,28]. TNFα was able to increase the self-renewal capacity of BCSC1 and BCSC2 by producing more mamosphere in a 3D sphere-forming assay. Similar effects were observed in a study where TNFα was able to increase the self-renewal of MCF-7 cells [39]. Additionally, TNFα is involved in tumor cell proliferation, invasion, and metastasis [40,41]. To address this, we exposed BCSC1 and BCSC2 to TNFα treatment and analyzed the cell growth. The cells proliferated exponentially in the presence of TNFα. Moreover, we showed an increased invasive and migratory capacity of BCSC1 and BCSC2 when treated with TNFα.

To date, there has been no data describing the angiogenic effects of TNFα in BC. In this study, we show that TNFα has a role in vascular attraction. Conditioned medium from BCSC1 cells pre-treated with TNFα showed an increase in the vascular network formation in vitro. Although the structure of tubules was significantly higher with the CM obtained from BCSC1, CM obtained from BCSC2 was unable to increase the tubules of HUVECs significantly. Nevertheless, the fact that TNFα being a pleotropic cytokine, could potentially have a distinct endothelial signaling cascade in BCSC2 and that could likely be a reason to miss recapitulate the similar effect of BCSC1 in network forming capacity.

Orthotopic transplantation of TNFα-treated BCSC2 showed increased tumor growth, while BCSC1 had homogenous tumor growth when compared to the untreated group. As mentioned previously, TNFα is a pleotropic cytokine and the fate of cells, such as survival, proliferation, or cell death, is determined by the intracellular signaling cascades induced by TNFα [42]. This data stipulates that the pleotropic effect on tumor growth induced by TNFα could be conceivable because of distinct downstream signaling pathways induced by TNFα in the two distinguishable cell lines.

Tumor-stroma interaction is the key player in tumor progression and the development of metastasis [43,44]. Our in vivo studies demonstrated that TNFα pre-treated BCSC1 and BCSC2 were able to enhance the capacity of the stromal cells to invade the primary tumor.

Our results indicate that enhanced collagen and fibronectin expression is required for the establishment of tumor-stromal invasion in the tumor microenvironment. This, in turn, enhanced the invasion of the endothelial cells towards the primary tumor. Studies reveal that the intravasation process occurs almost exclusively within the core of the primary tumor and, therefore, intravasation events are localized to the intra-tumoral angiogenic vasculature [31]. Synergetic interactions between the tumor, and stromal cells coalesce into abnormal organ-like structure that epitomize most human cancers, leading to tumor progression, including local invasions or the development of vascular niches to nurture the hematopoietic malignancies [45]. In congruence with the above statement, although the pleotropic effect of TNFα was notable in the progression of the tumor growth between the two distinguishable cell lines, the effect of TNFα in tumor-stromal interactions generating aberrant fibrotic septa in the primary tumor was consistent in both cell lines.

There is extensive evidence that modulations in the adhesive properties indulge in the malignancy of the tumor cells [16]. Tumor cells take advantage of the adhesion molecules to aid them in migration and homing during distant metastatic spread [46]. We demonstrate that TNFα increased VCAM-1 expression in the tumor tissue, which could be involved in the intra-tumoral stromal and endothelial invasion.

To our knowledge, we are the first group to investigate the effect of TNFα-induced breast-liver organ crosstalk, showing increased liver neovasculogenesis. Research indicates that cancer cells do not invade the secondary site passively and that these cells prime the host microenvironment or the pre-metastatic niche before the initiation of metastasis [47]. Kaplan et al. showed that hematopoietic progenitor cells (HPCs) enriched in VEGFR were able to form cellular clusters in the lung, priming the pre-metastatic niche before the tumor cell’s arrival [44,48]. It seems conclusive that the tumor cells primed for metastasis express certain genes, which could be clinically associated with gene expression in distant organs [44]. In line with the above statement and these observations, we show that TNFα-treated BCSCs showed an increase in the VCAM-1 expression in the primary tumor. Concomitantly, the liver neovasculogenesis was also enriched in the VCAM-1 expression. These data indicate that an inflammatory TNFα treatment primes the effects of BCSCs to secrete factors for preparing the distant pre-metastatic niche in the liver through VCAM-1 upregulation. We interpret the findings in the spirit of Paget’s “seed and soil theory” [49,50,51,52], wherein the TNFα-treated BCSCs (seed) prime the liver (soil) for the homing of metastatic cells.

However, the tumor cell’s adaptation to the milieu of a distant organ is a crucial and rate-limiting step in metastasis. Successful colonization of the metastatic cell varies broadly on the tumor type and organ [44] and metastasis is a complex multistep process [53]. Although our BCSCs stimulated with TNFα showed an increase in angiogenesis in the liver, which might be interpreted as a potential liver pre-metastatic niche under the stimulation of TNFα, in-depth further study of the underlying mechanisms and further extensive research is required to acknowledge successful liver metastasis.

In fact, there are many pathways considered as potential therapeutic targets to interfere with carcinogenesis. In particular, there is strong evidence that the deregulation of the nuclear-factor-κB (NF-κB) pathway is a major driver of inflammation that enhances cancer cell proliferation, metastasis, and resistance to several therapeutic treatments [54]. It further induces EMT, which facilitates distant metastasis. Therefore, it will be of fundamental interest to address the question if there is a direct crosstalk between TNFα and NF-κB signaling in promoting metastasis of BCSC to the liver tissue or in the development of new blood vessels within the tumor microenvironment in metastatic liver disease.

## 5. Conclusions

Overall, we identified TNFα as a potential angiogenic stimulus driving the pre-metastatic niche for homing metastatic cancer cells through VCAM-1 upregulation. Therefore, it would be prudent to focus more on the research of TNFα inhibition, but there are clearly pros and cons to this therapy [55]. New therapeutic strategies for TNFα signaling, including addressing NF-κB to target TNFα [56], may be of interest in the future. Our future work will strongly focus on the potential seed-and-soil effect in the liver elicited by TNFα potentially from chronic inflammation, to substantiate this potential link between the effect of TNFα on BCSCs and distant organ site reorganization [49,57]. For patients, it is of the utmost importance to detect metastasis early or even prohibit its occurrence. Our data may provide a valuable basis for future research to prevent the induction of a pre-metastatic niche and distant organ metastasis.

## Data Availability

The data that support the findings of this study are available from the corresponding author upon reasonable request.

## Supplementary Materials

The following supporting information can be downloaded at: https://www.mdpi.com/article/10.3390/biology11101481/s1, Table S1: Primers used for RT-qPCR; Table S2: Antibodies used for western blot analysis; Table S3: Antibodies used for immunohistochemistry; Table S4: Antibodies used for immunofluorescence staining; Table S5: VCAM-1 expression in the liver of mice. Supplementary Materials and Methods; Figure S1: Western blot of BCSCs; Figure S2: Keratin expression in TNFα-treated BCSCS; Figure S3: Migration assay of untreated and 10 days TNFα-treated BCSCs; Figure S4: Tube formation assay; Figure S5: Effects of TNFα-treated BCSC1 in vivo.; Figure S6: BCSCs show different signaling pathways; Figure S7: TNFα-treated BCSCs induced increased liver neovasculogenesis.

## Abbreviations

TNFα	Tumor Necrosis Factor-α
TNBC	Triple-Negative Breast Cancer
CSCs	Cancer Stem Cells
BCSCs	Breast Cancer Stem Cells
BC	Breast Cancer
ER	Estrogen Receptor
PR	Progesterone Receptor
EMT	Epithelial-To-Mesenchymal Transition
MEBM	Mammary Epithelial Basal Medium
MSC	Mammary Stem Cell
ACTB	Actin Beta
HUVECs	Human Umbilical Vein Endothelial Cells
EVG	Elastic Verhoeff–Van Gieson
VCAM-1	Vascular Cell Adhesion Molecule-1
