# Supplementary material for: Tumor Necrosis Factor-α (TNFα) Stimulate Triple-Negative Breast Cancer Stem Cells to Promote Intratumoral Invasion and Neovasculogenesis in the Liver of a Xenograft Model
Source: Biology (Basel). 2022 Oct 9;11(10):1481. doi: 10.3390/biology11101481 (PMC9598572; doi:10.3390/biology11101481)
Supplement: Supplementary file 1 [file biology-11-01481-s001.zip › biology-1931210-supplementary.pdf]

biology

MDPI

Supplementary Material

Supplementary Tables

Table S1. Primers used for RT-qPCR

Genes	Forward Primer	Reverse Primer
ACTB	CCAACCGCGAGAAGATGA	CCAGAGGCGTACAGGGATAG
E-cadherin	CCCGGGACAACGTTTATTAC	GCTGGCTCAAGTCAAAGTCC
EPCAM	CCATGTGCTGGTGTGTGAA	TGTGTTTTAGTTCAATGATGATCCA
Vimentin	GACCAGCTAACCAACGACAAA	GAAGCATCTCCTCCTGCAAT
Twist1	AGCTACGCCTTCTCGGTCT	CCTTCTCTGGAAACAATGACATC
Slug	TGGTTGCTTCAAGGACACAT	GCAAATGCTCTGTTGCAGTG
Snail	GCTGCAGGACTCTAATCCAGA	ATCTCCGGAGGTGGGATG

Table S2. Antibodies used for Western Blot Analysis

Antibody	Company
Anti-β-Actin	Sigma-Aldrich
Anti-E-Cadherin	BD Biosciences
Anti-Vimentin	GeneTex
Anti-Twist-1	Invitrogen
Anti-Slug	Cell Signaling

Anti-Snail	Cell Signaling
Rabbit anti-mouse IgG	Dako

Table S3. Antibodies used for Immunohistochemistry

**Antibody**	**Company**
Anti-Ki67	Abcam
Anti-Fibronectin	Abcam
Recombinant Anti-VCAM-1	Abcam
Anti-CD31	Invitrogen
Rabbit anti-mouse IgG	Dako

Table S4. Antibodies used for Immunofluorescence staining

**Antibody**	**Company**
Cytokeratin5 (K5)	Covance
Cytokeratin8 (K8)	Sigma-Aldrich
anti-mouse Alexa Fluor 568	Thermo Fisher
anti-rabbit Alexa Fluor 488	Thermo Fisher

Table S5. VCAM-1 expression in the liver of mice

**Cell Line**	**TNFα−**	**TNFα+**
BCSC1	0/5	3/5
BCSC2	0/3	2/3

Supplementary Materials and Methods

1 Immunofluorescence Staining

The keratin expression was determined using the Immunofluroscence staining. 96-well plates (Greiner Bio-One GmbH, Germany) were pre-coated with 50 µl Matrigel. 3×10^3 cells per well either untreated or TNFα-treated were seeded and allowed to reach 70% confluency. The cells were fixed with ice-cold methanol. The staining was conducted according to our previous study *Strietz, Stepputtis (1).* Listed antibodies were used (Additional file1: Table.S5). Images were taken using Invitrogen™ EVOS™ FL Auto Imaging System (ThermoFisher, USA).

2 Migration Assay

To analyse cell migration, cells untreated or treated with TNFα were seeded in 96-well plates at a density that reached confluence after 2 days of culture. Scratch wound per well was inflicted using the IncuCyte Wound Maker (Sartorius, Germany) according to the manufacturer's instructions. Scratch wound closure was monitored for 48 h by using the IncuCyte S3 Live-Cell Analysis System (Sartorius, Germany).

References

1. Strietz J, Stepputtis SS, Follo M, Bronsert P, Stickeler E, Maurer J. Human Primary Breast Cancer Stem Cells Are Characterized by Epithelial-Mesenchymal Plasticity. Int J Mol Sci. 2021;22(4).

Supplementary Figures

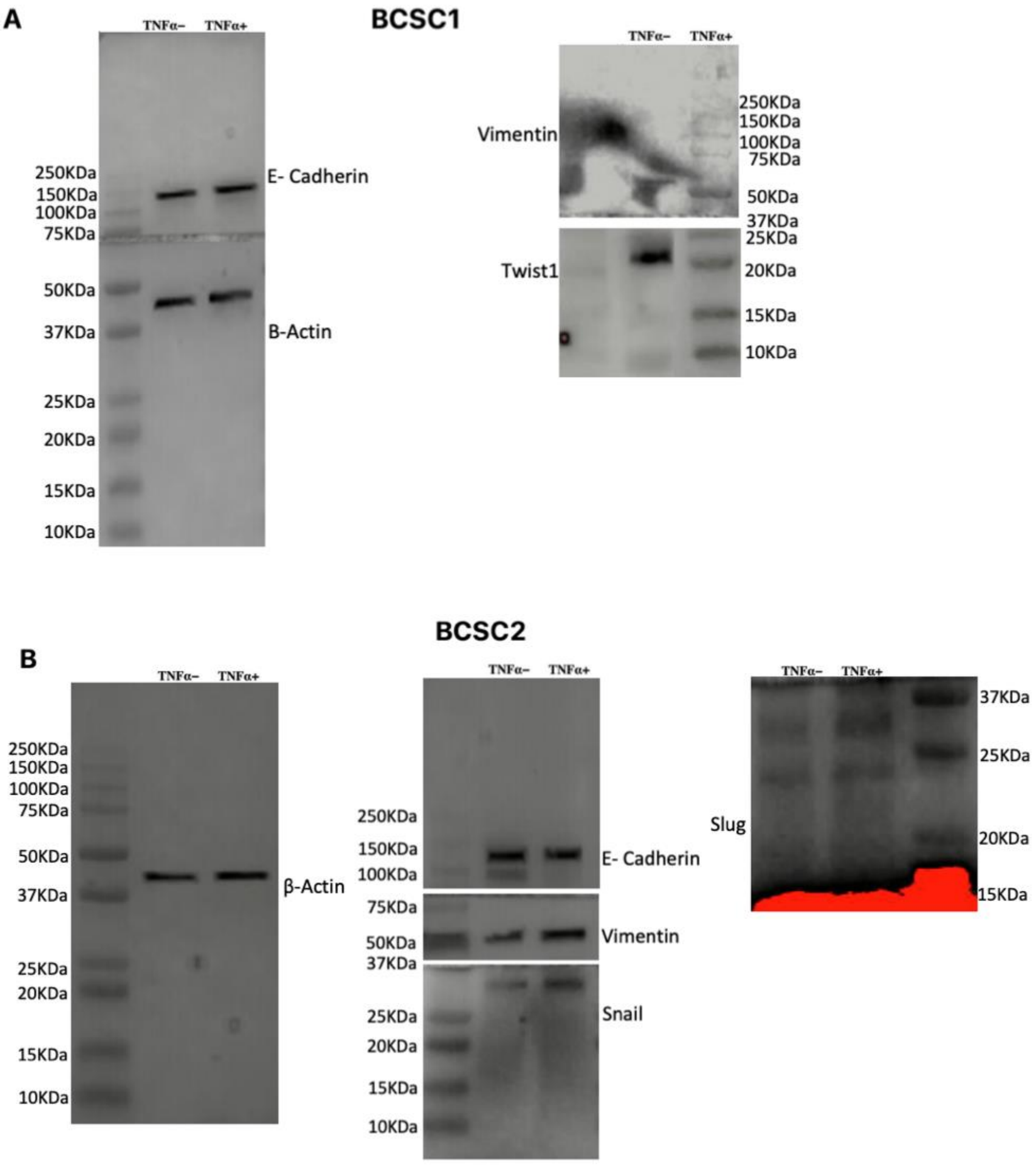

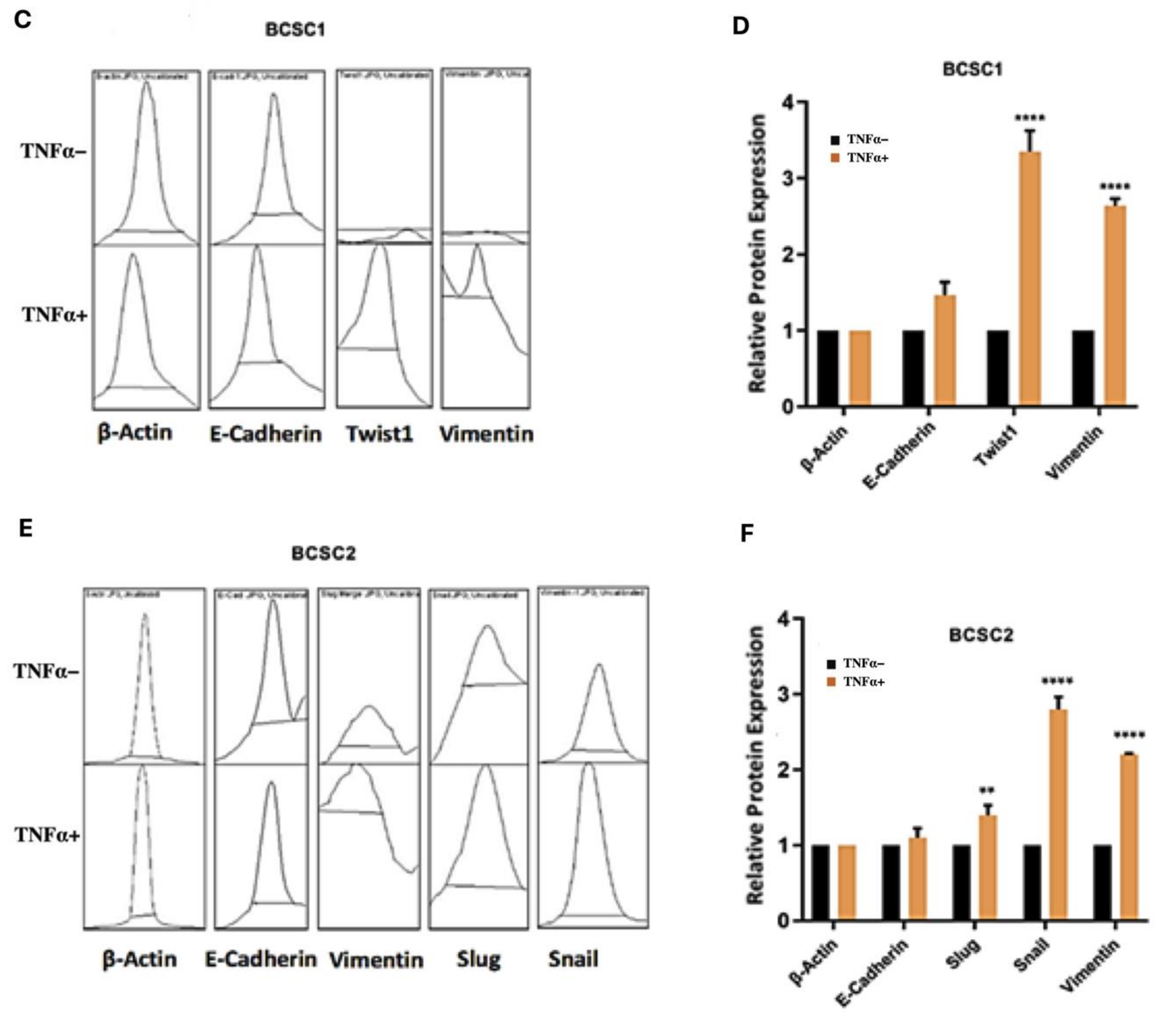

Figure S1. Western blot of BCSCs. (**A, B**) Original blot of BCSC1 and BCSC2. (**C-F**) Densitometric blot analysis of untreated and TNFα-treated BCSC1 and BCSC2. (**D, F**) Graphical representation of relative fold change of protein expression in BCSC1 and BCSC2 respectively (n=2). Data represents mean±SEM; ** $p<0.01$, **** $p<0.0001$ by 2-way ANOVA.

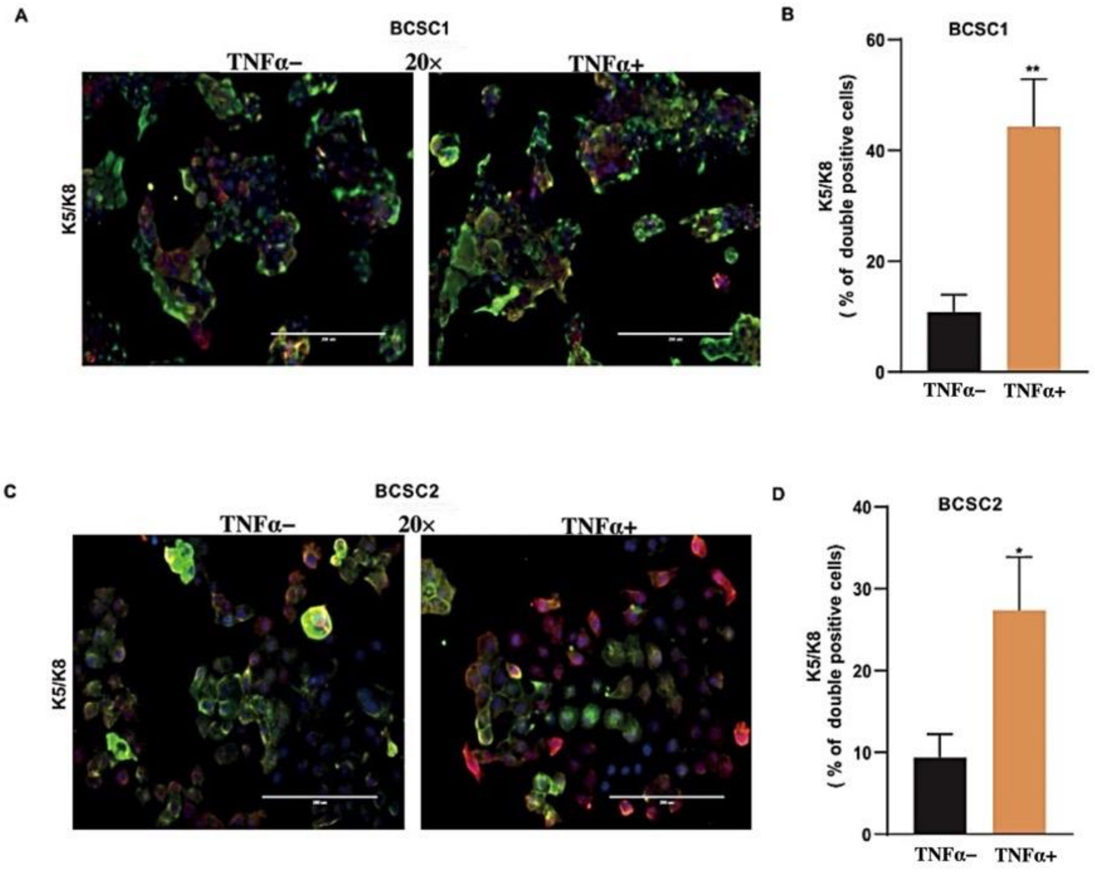

Figure S2. Keratin expression in TNFα treated BCSCS. (**A**) Keratin 5/Keratin 8 expression in BCSC1 untreated and TNFα-treated cells (n=2). Scale bars, 200 µm. (**B**) Graphical representation of keratin expression quantification in BCSC1 cells. (**C**) Keratin 5/Keratin 8 expression in BCSC2 untreated and TNFα treated cells (n=2). Scale bars, 200 µm. (**D**) Quantification of keratin expression in BCSC2 cells. Data represents mean±SEM; * $p<0.05$, ** $p<0.01$, by two-tailed, unpaired Student's *t-test.*

.

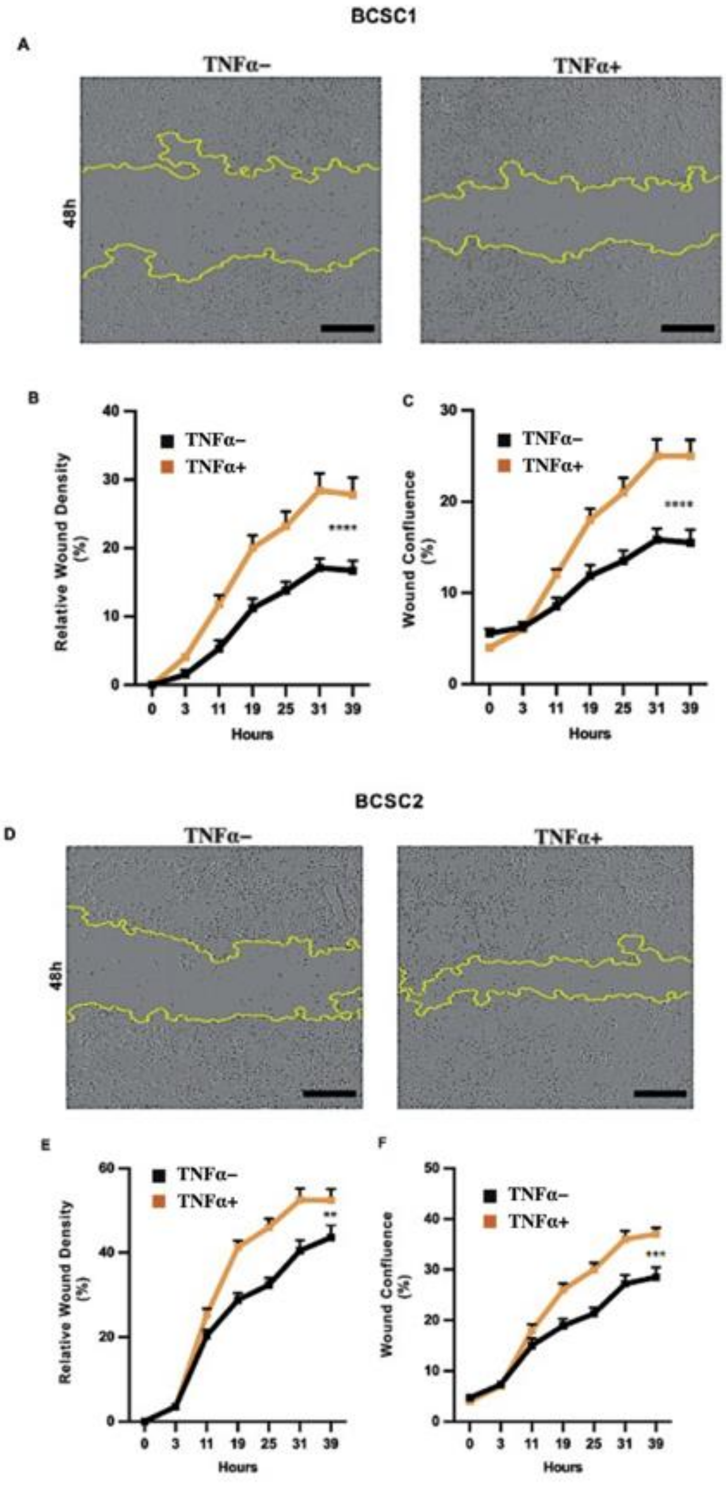

Figure S3. Migration assay of untreated and 10 days TNF*α*-treated BCSCs. (**A**) Images representing migration of BCSC1 at 48h when untreated (left) or treated with TNF*α* (right). Scale bars, 20 μm. (**B, C**) Quantification of relative wound density and wound confluence of BCSC1. (**D**) Images representing migration of BCSC2 at 48h when untreated (left) or treated with TNF*α* (right). Scale bars, 20 μm. (**E**,**F**) Quantification of

relative wound density and wound confluence for BCSC2. The experiment was performed once with 5 technical replicates. Data represents mean±SEM; ** p<0.01, *** p<0.001, **** p<0.0001 by 2-way ANOVA.

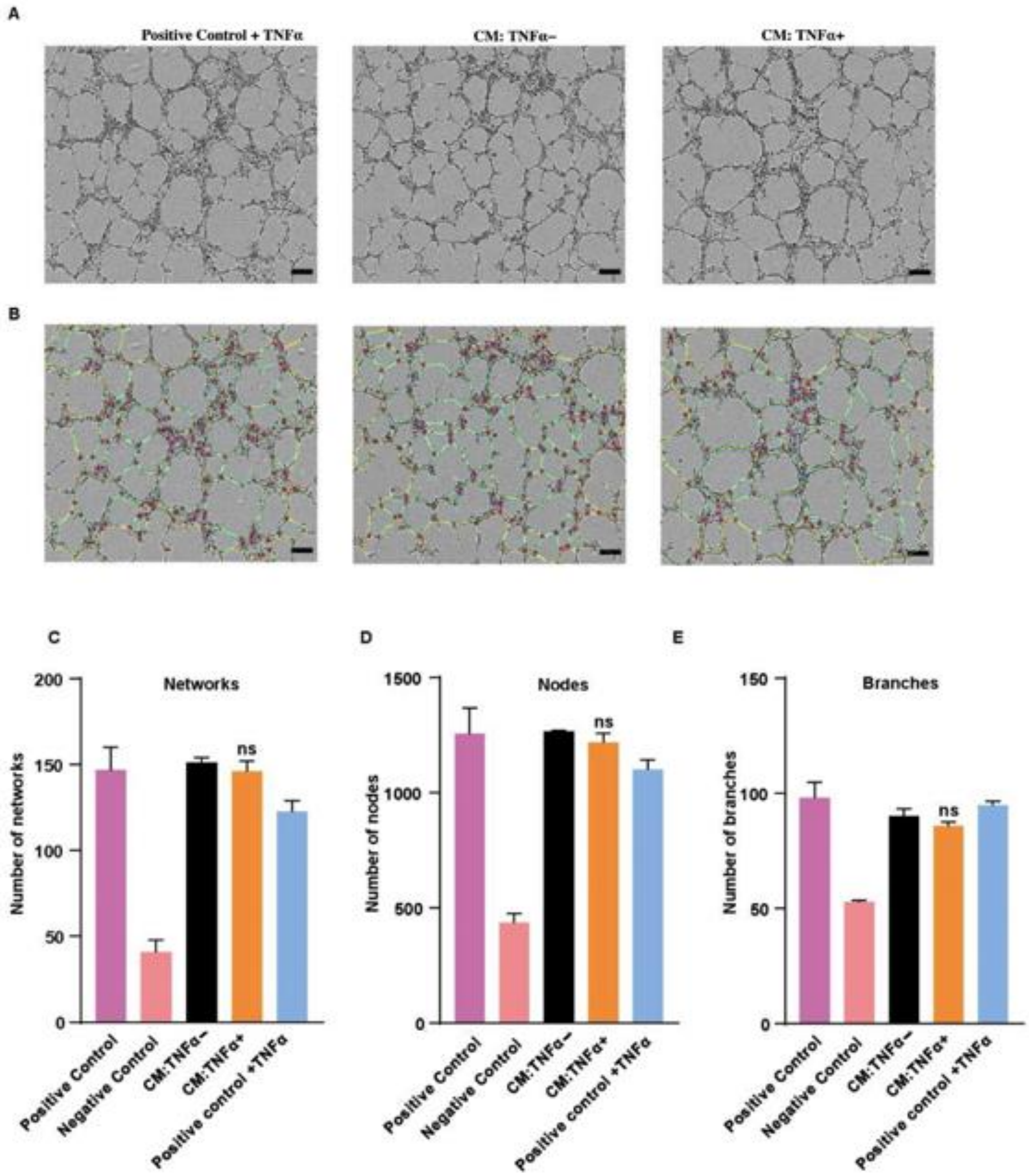

Figure S4. Tube formation assay of BCSC2. (**A**) TNFα-CM from BCSC2 failed to promote the tube formation of HUVECSs *in vitro*. Scale bars, 15 μm. (**B**) Representative images depicting the quantitative analysis of the tube formation. Data analysis was performed with Image-J Plugin Angiogenesis Analyser. Blue colour indicate the networks. Small circles indicate the nodes. Green colour indicates the branches. (**C**,**D, E**) Graphical data demonstrating the number of networks, nodes and branches (n=2). Data represents mean±SEM; ≥ 0.05, ns, by 1-way ANOVA.

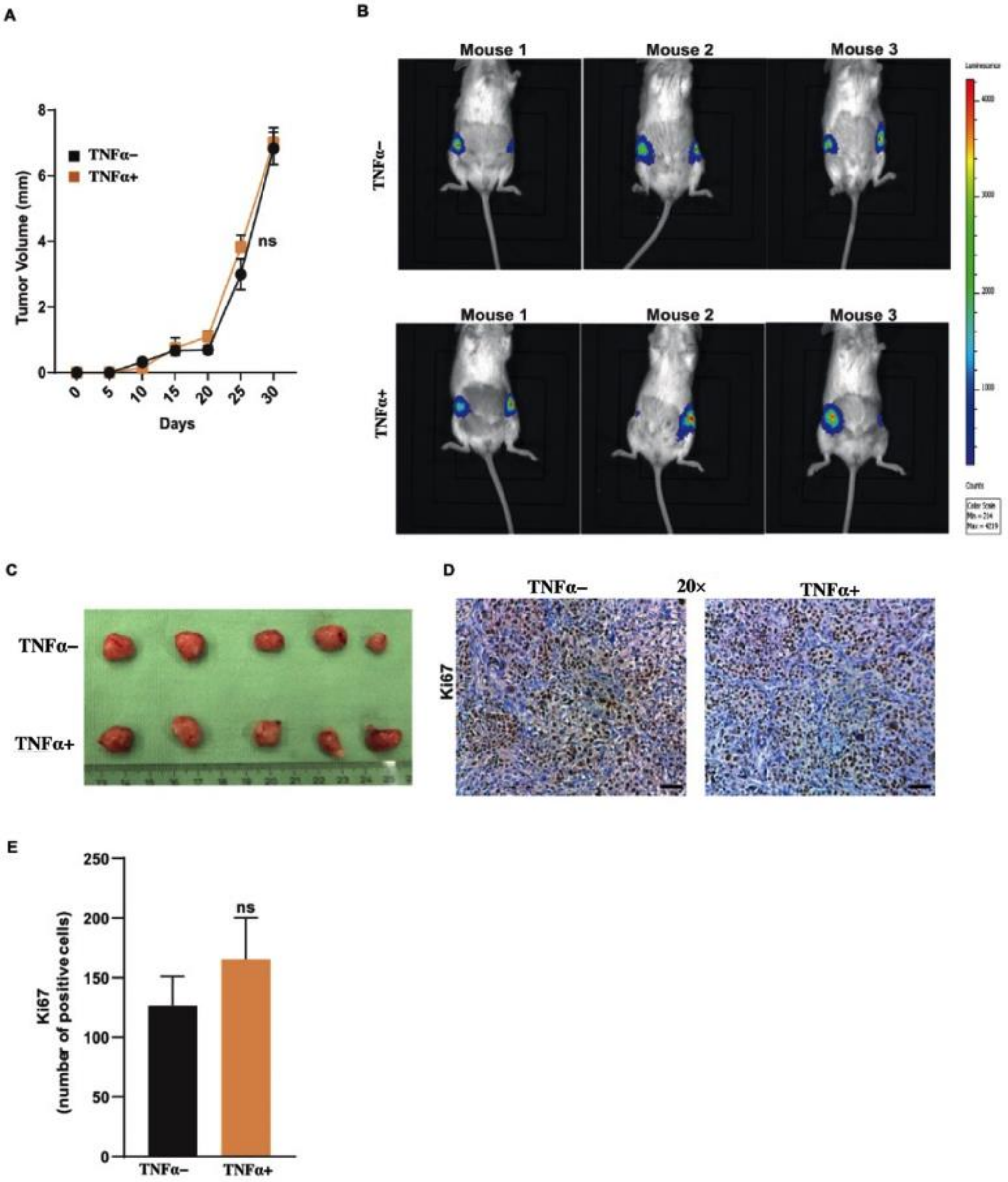

Figure S5. Effects of TNFα-treated BCSC1 *in vivo*. (**A**) Tumor growth curve of BCSC1 over time (n=6). Data represents SD ± SEM; ≥ 0.05, ns, by 2-way ANOVA. (**B**) Representative bioluminescent images of the tumor-bearing mice 3.5 weeks post transplantation. (**C**) Excised tumors of the mice from the two experimental group (n=6). (**D**) Ki67 staining of the tumor tissues (n=4). Scale bars, 15 μm. E**,** Mice that received TNFα treated BCSC1 showed no significance in regard to Ki67 staining compared to the untreated BCSC1. Data represents mean±SEM; ≥ 0.05, ns, by two-tailed, unpaired Student's *t-test*.

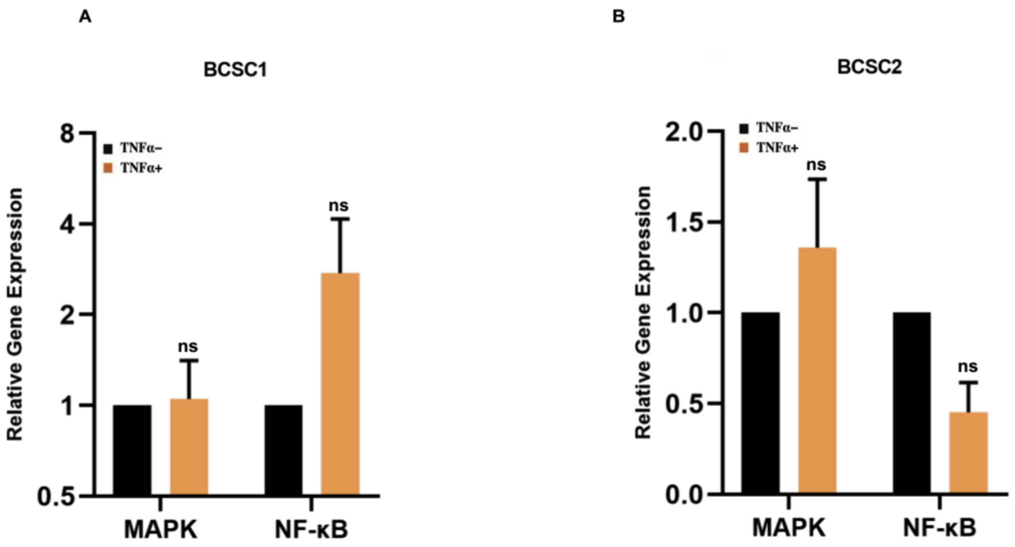

Figure S6. BCSCs show different signaling pathways. Tumors were excised and subjected to RNA isolation. Primary gene expression analysis of the signaling pathway was performed**.** (**A**) Tumors from mice that received TNFα-treated BCSC1 showed increase in NF-κB pathway (n=3). (**B**) Tumors from mice that received TNFα-treated BCSC2 showed increase in MAPK pathway (n=4). All Data represents mean±SEM; ≥ 0.05, ns, by 2-way ANOVA.

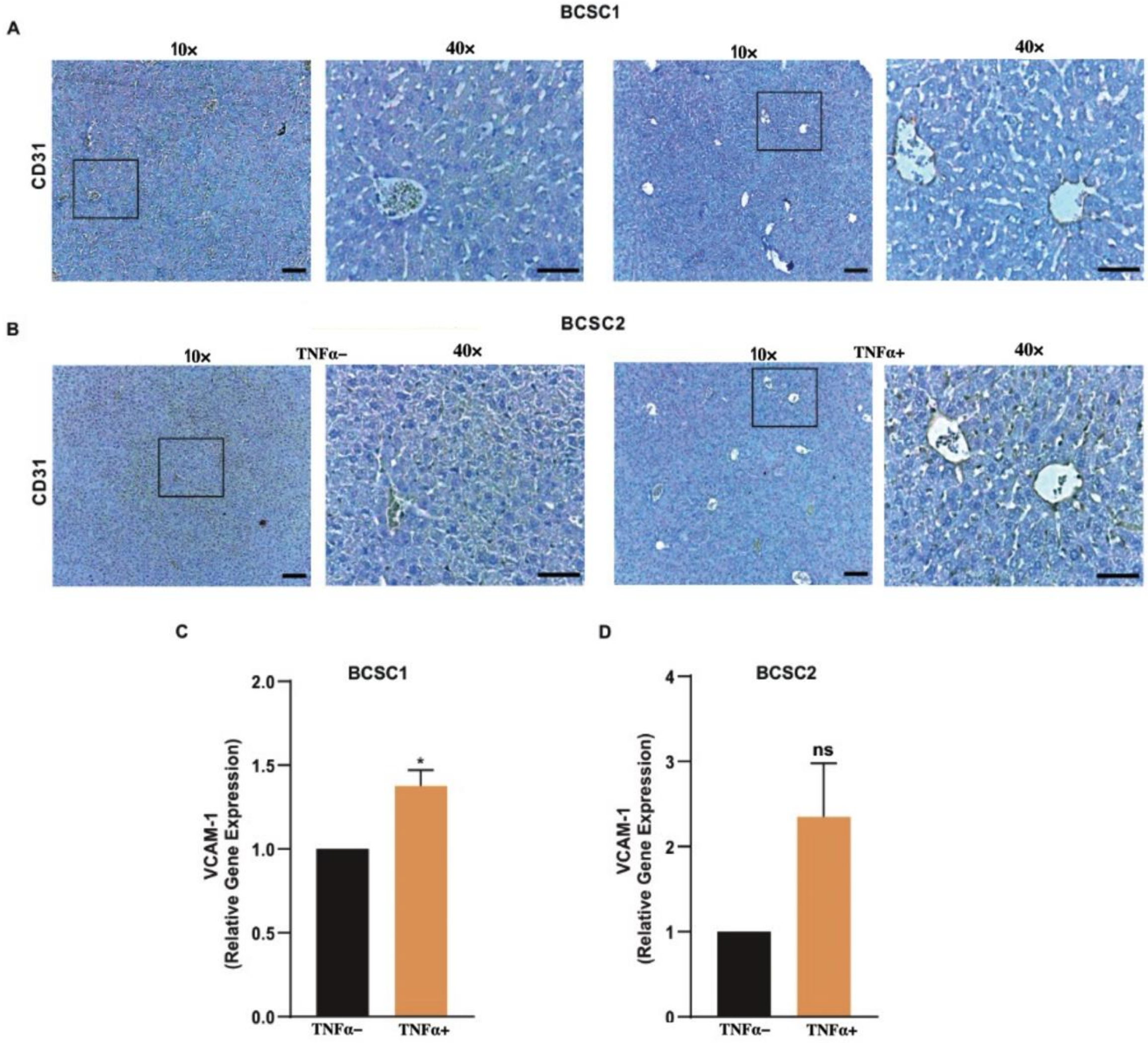

Figure S7. TNFα-treated BCSCs induced increased liver neovasculogenesis. (**A**) Representative images depicting the CD31 staining of the mice liver tissue indicating low CD31 expressing blood vessels in untreated BCSC1 (left) (n=3) and increase in CD31 positive blood vessels in the TNFα treated BCSC1 (right) (n=3). (**B**) CD31 staining in the untreated BCSC2 (left) (n=3) and TNFα-treated BCSC2 (right) (n=3) group. Scale bars, 30 µm and 15 µm. (**C, D**) Quantification of VCAM-1 mRNA expression isolated from the liver tissue from BCSC1 and BCSC2 cells respectively (n=3). Data represents mean±SEM; ≥ 0.05, ns, * p<0.05, by two-tailed, unpaired Student's *t-test.*
